# eHealth Self-Management Interventions for Patients With Liver Cirrhosis: Scoping Review

**DOI:** 10.2196/68650

**Published:** 2025-09-10

**Authors:** Seojin Lee, Youngshin Joo, Yeonsoo Jang

**Affiliations:** 1 College of Nursing and Brain Korea 21 FOUR Project, Yonsei University, Seoul, Republic of Korea; 2 Mo-Im Kim Nursing Research Institute, Institution for Innovation in Digital Healthcare, College of Nursing, Yonsei University, 50-1 Yonsei-ro, Seodaemun-gu, Seoul, Republic of Korea

**Keywords:** digital health intervention, liver cirrhosis, self-management, health behavior change, eHealth, scoping review, PRISMA

## Abstract

**Background:**

Liver cirrhosis (LC) is a morbid condition associated with frequent hospitalization and high mortality. Effective self-management is essential for patients with LC to monitor fluctuating symptoms and follow complex treatment regimens. However, strategies are often unsustainable and insufficiently tailored to individuals with cognitive impairments. Although eHealth interventions enable continuous monitoring, personalized guidance, and improved accessibility in other chronic conditions, comprehensive evidence for eHealth self-management interventions specifically tailored to patients with LC remains limited.

**Objective:**

This scoping review systematically identified and described existing eHealth self-management interventions for patients with LC, analyzed their core components, and summarized the reported outcome variables.

**Methods:**

Five electronic databases (PubMed, Embase, Cochrane Library, CINAHL, and Web of Science) were systematically searched for studies published between 2013 and June 2025. Interventional studies targeting adults with LC and involving eHealth-based self-management were included. Data on study design, intervention components, delivery methods, and outcome domains were extracted. The results were synthesized descriptively using the Arksey and O’Malley framework, and reporting followed the PRISMA-ScR (Preferred Reporting Items for Systematic Reviews and Meta-Analyses Extension for Scoping Reviews) guidelines.

**Results:**

Among 19,695 records screened, 9 studies met the inclusion criteria, comprising 1 randomized controlled trial, 2 quasi-experimental studies, 1 pilot test, 2 feasibility studies, 2 usability studies, and 1 cost-effectiveness study. The 8 intervention studies utilized smartphone apps or telephone and incorporated 6 key components: symptom monitoring (100% of the included studies), lifestyle behavior modification (n=5, 63%), information provision (n=5, 63%), alert-triggered responses (n=3, 38%), counseling and motivation (n=2, 25%), and reminders (n=2, 25%). The intervention durations ranged from 1 to 6 months. Among all 9 studies, outcomes were assessed across 5 domains: physical (n=3, 33%), psychosocial (n=2, 22%), clinical (n=4, 44%), self-management (n=3, 33%), and implementation (n=6, 67%). Some studies reported improvements in hospital admissions (n=4, 44%), muscle mass (n=1, 11%), self-management knowledge (n=1, 11%), and cost outcomes (n=1, 11%).

**Conclusions:**

This review identified diverse eHealth self-management interventions with core components for patients with LC, evaluated across multiple outcome domains. Nonetheless, the evidence remains limited by small sample sizes and heterogeneous study designs and outcome measures. Future research should prioritize rigorous randomized trials, standardized intervention frameworks, and core outcome sets to support clinical implementation and effectiveness evaluation.

## Introduction

### Background

Liver disease accounts for approximately 4% of all global deaths, with liver cirrhosis (LC) ranking as the 15th leading cause of disability-adjusted life-years worldwide [1]. Despite a modest decline in cirrhosis-related mortality in recent years, liver disease remains a major public health concern in South Korea [2-4].

LC is a progressive condition resulting from chronic liver damage caused by hepatitis B or C infection, metabolic dysfunction-associated steatotic liver disease (MASLD), excessive alcohol consumption, or metabolic syndrome [5-9]. Disease progression can lead to serious complications such as ascites, variceal bleeding, and hepatic encephalopathy [10-13], which necessitate frequent hospitalizations and increased health care costs [8,13-15] and significantly impair the patient’s quality of life (QoL) [16-19].

LC requires continuous self-management [20], involving patients’ ability to maintain their health and manage the effects of their illness in everyday life [21-23]. This is particularly challenging due to unpredictable symptoms; cognitive impairment; and the complexity of managing multiple lifestyle modifications such as dietary control, regular exercise, alcohol cessation, weight management, and medication adherence [5,23,24].

Although evidence from other chronic diseases demonstrates that self-management interventions can improve symptom control and reduce hospitalizations [22,25], evidence in LC remains limited and mixed. Although some benefits have been reported, including improved self-management behaviors, symptom awareness, and reduced hospital readmissions [24,26], traditional approaches face significant limitations, including patient cognitive difficulties owing to hepatic encephalopathy, as well as limited scope and poor sustainability of the interventions [16,17].

To address these limitations, eHealth interventions using digital technologies such as mobile apps, web-based platforms, and remote monitoring devices [27] have emerged as promising solutions. These platforms offer continuous monitoring, personalized interventions, and improved accessibility [28-32]. eHealth interventions in chronic diseases have demonstrated improved self-management behaviors, reduced hospitalizations, and enhanced patient outcomes [33-35]. eHealth interventions for individuals with LC aim to support patient self-monitoring and disease management and have demonstrated potential applications in symptom tracking and therapeutic support [36,37]. Although individual eHealth interventions for patients with LC have been explored, a comprehensive synthesis of existing evidence is required to understand their current state and potential.

Therefore, this scoping review systematically identified and described existing eHealth-based self-management interventions for patients with LC, examined their key components, and summarized their outcome variables.

### Objectives

This review aimed to provide foundational knowledge to support the development of effective and accessible eHealth self-management interventions in clinical practice by exploring current evidence on eHealth self-management interventions for patients with LC. Specifically, the review focused on identifying the characteristics of relevant studies, examining the types and core components of the relevant interventions, and summarizing the health outcome variables reported in the literature.

This review was guided based on the following research questions:

1. What are the characteristics and research designs of studies examining eHealth self-management interventions for patients with LC?
2. What are the contents and core components of these interventions?
3. What health outcomes have been assessed, and what findings have been reported across the included studies?

## Methods

This scoping review followed the Arksey and O’Malley framework [36,38], with enhancements from the Joanna Briggs Institute (JBI) methodology for scoping reviews [39]. Reporting adhered to the PRISMA-ScR (Preferred Reporting Items for Systematic Reviews and Meta-Analyses Extension for Scoping Reviews) guidelines [40].

### Eligibility Criteria

The inclusion criteria were developed based on the Population, Concept, Context (PCC) framework recommended by the JBI methodology for guiding scoping reviews. The criteria were structured as follows:

1. Study population: adults (aged ≥18 y) diagnosed with LC
2. Concept: interventions delivered through eHealth, including but not limited to mobile health, web-based platforms, mobile apps, or telehealth services, that support self-management activities such as symptom monitoring and management, medication adherence, or lifestyle modification related to diet, physical activity, alcohol consumption, or smoking
3. Context: clinical or community health care settings

We included all interventional studies reporting at least one outcome related to the implementation, utilization, or impact of eHealth-based self-management interventions. Only peer-reviewed full-text articles were included, according to the search strategy.

Studies were excluded if they (1) did not report any outcomes (eg, review papers, study protocols, commentaries, editorials, or conceptual papers); (2) were purely qualitative, without presenting any outcome findings; (3) applied treatment-based interventions that focused only on pharmacological or invasive procedures; (4) focused on diagnostic or screening tools such as computed tomography or magnetic resonance imaging; and (5) were not published as peer-reviewed full-text articles (eg, conference abstracts, preprints, conference proceedings, or letters to the editor).

### Information Sources

We systematically searched the following five electronic bibliographic databases: PubMed, Embase, Cochrane Library, CINAHL, and Web of Science. The search was performed in June 2025 and included all studies indexed up to that date. No restrictions were placed on geographic location. Language was restricted to English and Korean, given the linguistic capabilities of the review team.

To supplement the database search, we manually screened the reference lists of the included studies and relevant review articles and conducted forward citation tracking using Google Scholar. These supplementary searches did not identify any additional eligible studies.

### Search Strategy

The search strategy, developed in collaboration with a medical librarian starting in June 2025, focused on key concepts related to LC, eHealth interventions, and self-management. The initial search terms were developed based on the PCC framework, incorporating both controlled vocabulary (eg, Medical Subject Headings [MeSH] in PubMed, Emtree in Embase, and CINAHL subject headings) and free-text keywords. A medical librarian with expertise in health sciences literature assisted in refining the search strategy to ensure its sensitivity and relevance across databases. Two researchers with prior experience in evidence synthesis (Y Joo and Y Jang) independently reviewed and optimized the search terms and Boolean logic.

Preliminary searches were conducted to inform term selection and refine the strategy. The final search strategy included the terms (“liver cirrhosis” OR “liver disease”) AND (“self-management” OR “self-care” OR “lifestyle modification”) AND (“eHealth” OR “mHealth” OR “telehealth”). The full search strategies for each database are detailed in Multimedia Appendix 1.

### Study Selection

All identified records were imported and compiled into EndNote X21 [41] for reference management. Duplicates were initially removed using EndNote’s automatic tool; additional duplicates were identified by manually checking based on the titles, authors, and publication years in Microsoft Excel LTSC Professional Plus 2021 (Microsoft Corporation).

Two reviewers (SL and Y Joo) independently screened the titles and abstracts of all retrieved records against the predefined inclusion and exclusion criteria. Full-text articles of studies deemed potentially eligible were then assessed independently by the same reviewers. Any disagreements regarding study inclusion were resolved through discussion, with a third reviewer (Y Jang) consulted when a consensus could not be reached. The final selection was made by consensus of the three researchers. The screening process was conducted according to the JBI methodology for scoping reviews.

### Data Charting

Two researchers (SL and Y Joo) independently charted and cross-checked the data from the included studies using a standardized Excel form developed by the research team. The extracted information comprised study characteristics (eg, authors, year, country, study design), presence of control groups or comparisons, details about the eHealth self-management intervention, and outcome variables. The charting form was developed a priori based on the PCC framework and refined through team discussion. Two reviewers (SL and Y Joo) independently assessed the extracted data for consistency. Any discrepancies were resolved through discussion, with input from a third reviewer (Y Jang) when necessary.

### Data Analysis and Synthesis

The extracted data were synthesized using a narrative synthesis and organized into 3 main sections. The first section summarized the characteristics of the included studies, including authors, publication year, country, study design, participants, sample size, and participant age. The second section focused on the eHealth interventions’ contents and delivery, including intervention type, delivery mode, and providers. The third section described the implementation details and outcomes, including data collection methods, intervention duration, outcome variables, and key findings. Due to the heterogeneity of the study designs and reported outcomes, meta-analysis was not feasible. Therefore, the findings are presented narratively and summarized in tables.

## Results

### Study Selection

**Figure 1.**
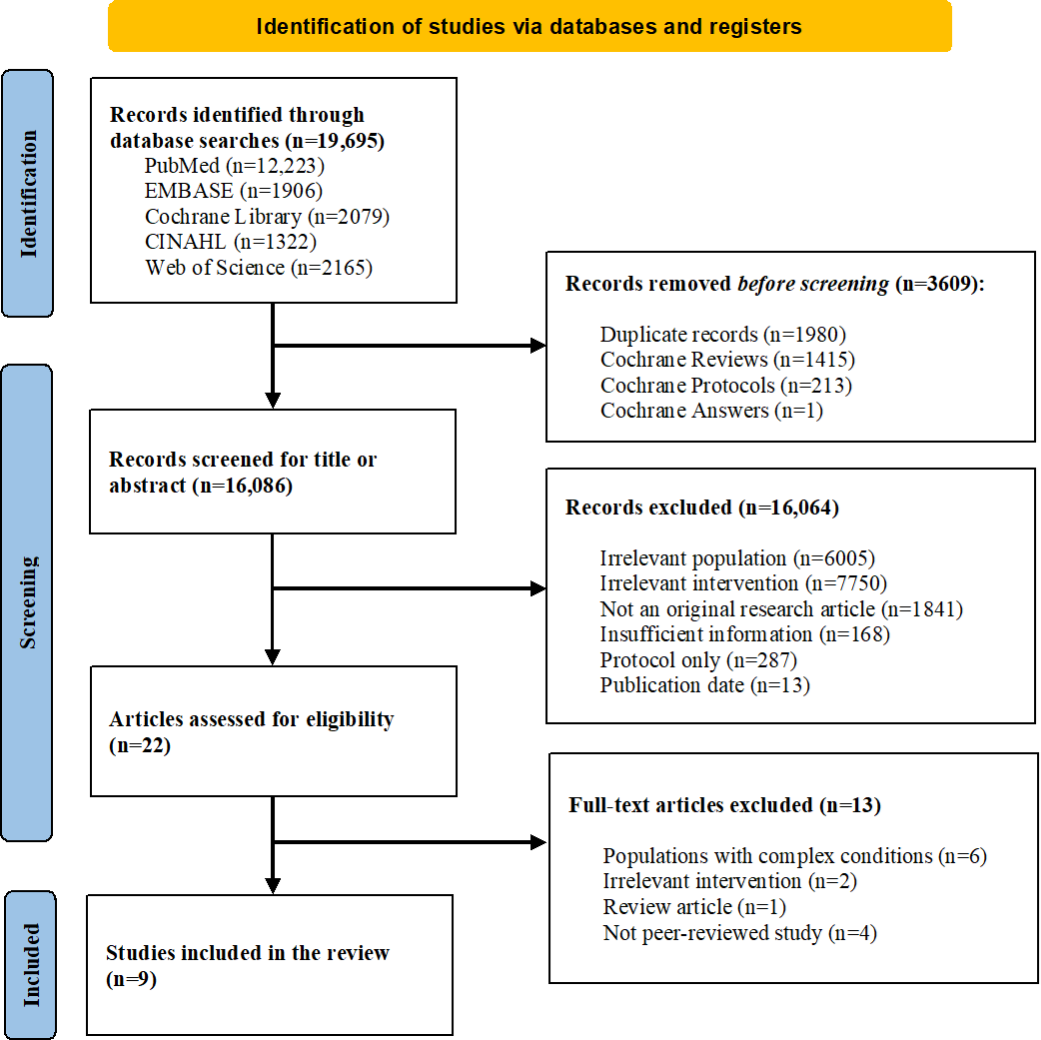
PRISMA-ScR (Preferred Reporting Items for Systematic Reviews and Meta-Analyses Extension for Scoping Reviews) flow diagram of the study selection process.

The initial search identified 19,695 records from 5 relevant databases. After removing 1980 duplicate records and 1629 Cochrane reviews, protocols, and answers, 16,086 records were included in the title and abstract screening. The study selection process is illustrated in the PRISMA-ScR flow diagram (Figure 1). A total of 16,064 records were excluded for irrelevant populations, irrelevant interventions, and insufficient information, which were identified through manual review. Additionally, we excluded records that were not available as full texts, such as abstracts and e-posters. We then assessed the full texts of 22 potentially eligible papers, 13 of which were excluded for including participants with complex multimorbidity not limited to liver disease, reporting irrelevant interventions, including irrelevant article types (reviews), and lacking peer review. Finally, 9 studies met the inclusion criteria and were included in the present review [36,42-49].

### General Characteristics of the Included Studies

The general characteristics of the included studies are summarized in Table 1. All studies were published between 2019 and 2024, with nearly half (n=4, 44%) published in 2024. The studies were published in various countries, with the largest number of studies being published in the United States (n=3, 33%). The studies employed diverse research designs, including 1 (11%) randomized controlled trial (RCT) [46], 2 (22%) quasi-experimental studies [47,49], 1 (11%) pilot test study [43], and 2 (22%) feasibility test studies [36,45]. Additionally, 2 (22%) studies applied application usability tests [44,48], and 1 (11%) study conducted a cost-effectiveness analysis [42].

Regarding study design, 5 (56%) studies included control or comparison groups [42,43,46,47,49], whereas the remaining 4 (44%) studies used a single-group pre-post design [36,44,45,48]. The controlled studies included designs with historical controls, RCTs, multigroup comparisons, and model-based analyses, while the single-group studies focused primarily on feasibility and usability evaluation.

Sample sizes in the intervention studies ranged from 18 to 124 participants, with 4 (44%) studies having <50 participants. The mean age was 56.3 (SD 10.6) years [36,43-47,49], and 1 study reported age using a categorical range (20-60 y), with >70% of participants <40 years of age [48]. Another study used a decision analytic simulation model based on a hypothetical cohort of 100 patients and did not include actual participants [42].

All 8 studies with sample sizes used mobile apps to deliver interventions. Five (63%) studies relied solely on mobile apps without telephone contact [44-46,48,49]. Five (63%) studies integrated Bluetooth-enabled devices, 3 (38%) of which also used telephone contact to support clinical decision-making.

**Table 1.** General characteristics of the included studies (N=9).

Features	Studies	References
Country of publication, n (%)		
United States	3 (33)	[36,42,47]
Iran	2 (22)	[44,48]
Canada	1 (11)	[45]
China	1 (11)	[49]
Taiwan	1 (11)	[46]
United Kingdom	1 (11)	[43]
Study design, n (%)		
Randomized controlled trial	1 (11)	[46]
Quasi-experimental	2 (22)	[47,49]
Pilot test	1 (11)	[43]
Feasibility test	2 (22)	[36,45]
Usability test	2 (22)	[44,48]
Cost-effectiveness	1 (11)	[42]
Sample size, n (%)		
<50	4 (44)	[36,43,45,48]
50-99	2 (22)	[44,46]
≥100	2 (22)	[47,49]
N/A^a^ (model-based simulation)	1 (11)	[42]
Mode of delivery, n (%)^b^		
Mobile app only	5 (56)	[44-46,48,49]
Mobile app and telephone call	3 (33)	[36,43,47**]**
Participant’s age (y)		
Studies, n (%)	7 (78)	[36,44-49]
Mean (pooled SD)	56.3 (10.6)	[36,44-49]

a N/A: not applicable.

b Bloom et al [42] was excluded from this categorization because it was a model-based economic evaluation study derived from the intervention conducted by Bloom et al [36].

### Components of the eHealth Self-Management Interventions

Among the 9 included studies, 8 (89%) implemented eHealth interventions for patients with LC [36,43-49], while 1 study conducted a model-based cost-effectiveness analysis without directly implementing an intervention [42]. As that study performed an economic evaluation based on the feasibility trial reported by Bloom et al [36], it was excluded from the intervention component analysis.

The remaining 8 intervention studies addressed a range of self-management components. Table 2 outlines 6 key components commonly incorporated in these eHealth programs for patients with LC: (1) symptom monitoring (n=8, 100%), (2) health behavior modification (n=6, 75%), (3) information provision (n=5, 63%), (4) counseling and motivation (n=7, 88%), (5) alert-triggered intervention (n=4, 50%), and (6) reminders (n=4, 50%).

**Table 2.** Components of the self-management interventions described in the included studies (n=8). Note: Bloom et al [42] was excluded from this table because it was a model-based economic evaluation study derived from the intervention conducted by Bloom et al [36].

Components	Studies, n (%)	References
Symptom monitoring		
Physical (vital signs, weight)	8 (100)	[36,43-49]
Cognitive status	3 (38)	[43,45,48]
Psychological (well-being)	2 (25)	[43,45]
Health behavior modification		
Dietary	5 (63)	[36,43,45,47,49]
Medication adherence	3 (38)	[46,47,49]
Physical activity	2 (25)	[45,49]
Information		
Physical activity	5 (63)	[44-46,48,49]
Disease information	3 (38)	[44,46,48]
Nutrition	3 (38)	[44,45,48]
Counseling and motivation		
Individual expert counseling	2 (25)	[45,47]
Decision support	2 (25)	[36,43]
Q&A	2 (25)	[44,48]
Case sharing (motivational support)	1 (13)	[46]
Alert-triggered intervention		
Referral or follow-up care	3 (38)	[36,43,47]
GPS-based location sharing	1 (13)	[48]
Reminder		
Daily measurement	2 (25)	[36,43]
Medication	2 (25)	[46,48]

#### Symptom Monitoring

Symptom monitoring was the most commonly implemented component across the included studies and was reported in the interventions described in all 8 (100%) studies [36,43-49]. The monitoring covered physical, cognitive, and psychological domains using self-reported and self-measured formats. Physical symptom monitoring was the most prevalent, including indicators such as blood pressure, heart rate, and body weight (n=8, 100%) [36,43-49]. Three (38%) studies reported cognitive symptom monitoring, using structured questionnaires to assess changes in attention, memory, or cognitive function [43,45,48]. Two (25%) studies reported interventions that monitored psychological symptoms to address well-being [43,45].

#### Health Behavior Modification

Interventions targeting health behavior most frequently focused on dietary management, followed by medication adherence and physical activity. Dietary management was implemented in 5 (63%) studies [36,43,45,47,49] and included strategies such as optimizing protein intake, restricting sodium, setting behavioral goals, and supporting meal preparation. Three (38%) studies included support for medication adherence, typically through reminder systems or self-reporting features [46,47,49]. Physical activity components were incorporated in 2 (25%) studies, including home-based exercise [45,49].

#### Information

Educational content included information on physical activity, nutrition, and disease-related knowledge. Physical activity education was the most frequently reported (n=5, 63%) [44-46,48,49], followed by disease-related (n=3, 38%) [44,46,48] and nutrition (n=3, 38%) [44,45,48] education. The delivery methods for educational and motivational content varied and included videos, interactive modules, or multimedia formats [44-46,48,49].

#### Counseling and Motivation

Interventions addressing counseling and motivation were implemented in various forms. Individual expert counseling services were described in 2 (25%) studies [45,47], whereas the interventions described in 2 (25%) studies applied clinical decision support systems, which allowed health care providers to review symptom or weight data and adjust treatments accordingly [36,43]. Additionally, Q&A features were available in 2 (25%) interventions, which enabled patients to ask health-related questions through the platform [44,48]. One (13%) study described an intervention that presented motivational case stories to enhance users’ confidence in making lifestyle changes.

#### Alert-Triggered Interventions

Alert-triggered functions were incorporated in the interventions included in 3 (38%) studies [36,43,47], in which automated notifications informed health care providers of clinical deterioration (eg, weight gain or symptom exacerbation), allowing timely follow-up care. Additionally, 1 (13%) study included GPS-based location sharing to allow patients to notify caregivers of their physical condition and location during acute events [48].

#### Reminders

Reminder systems were essential tools to reinforce patient adherence to self-management routines. Two (25%) studies reported on interventions that used daily prompts to encourage consistent weight or symptom tracking [36,43]. Another 2 (25%) studies provided medication reminders through app-based notifications [46,48].

#### Intervention Providers and Duration

The interventions were delivered by a range of provider types across the included studies. Three (38%) studies employed a collaborative model involving physicians and nurses as intervention providers [36]. One (13%) study reported a physician-led intervention [43], while 2 studies reported primarily nurse-led interventions, in which nurses monitored patient data, provided education, or delivered the intervention directly [46,47]. A multidisciplinary team consisting of a dietitian and an exercise specialist delivered the intervention in another study [45]. Three studies did not explicitly report the provider type involved in the intervention delivery [44,48,49].

The intervention durations ranged from 1 to 6 months. One study implemented a 6-month intervention [48], while 4 studies employed interventions lasting approximately 3 months [43,45,47,49]. Two (25%) studies used 1-month interventions [36,46], and 1 (13%) study did not clearly report the duration [44].

### Health Outcome Variables

The health-related outcome variables and study-specific findings are summarized in Table 3. The 9 included studies assessed a wide range of outcome domains in 4 categories: (1) physical outcomes (n=3, 33%), (2) psychosocial outcomes (n=2, 22%), (3) clinical outcomes (n=4, 44%), and (4) self-management outcomes (n=3, 33%).

**Table 3.** Summary of health-related outcome variables and results reported (N=9).

Variables	Results	References
Physical		
Muscle mass and sarcopenia	Improved^a^	[49]
Physical function	Improved^b^	[45]
Nutritional biomarkers	Mixed^a^	[49]
Disease severity	Improved ^a^	[43]
Unplanned large-volume paracentesis	Decreased^a^	[43]
Psychosocial		
Cognitive status	Improved^a^^,b^	[45,47]
Quality of life	No improvement^b^	[45]
Clinical		
Hospital admissions	Reduced^a^^,c^	[36,42,43,47]
Mortality	Mixed^a^	[43,47]
Self-management		
Physical activity behavior	Improved^a^	[45,49]
Diet management	Improved^b^	[45,46]
Self-management practice score	Improved^a^	[46]
Self-management knowledge score	Improved^a^	[46]

a Based o then comparison between intervention and control groups.

b Based on pre-post comparison in the intervention of a single group (no control group).

c Reported from a model-based simulation study.

#### Physical Outcomes

The assessment of physical outcomes focused on 5 key domains: muscle mass and sarcopenia, physical function, nutritional biomarkers, disease severity, and unplanned large-volume paracentesis [43,45,49]. One (11%) study evaluated muscle and sarcopenia outcomes based on sarcopenia prevalence, skeletal muscle index, and grip strength in patients receiving a walking exercise program combined with branched-chain amino acid supplementation [49]. Another study assessed physical function using the liver frailty index and 6-minute walk test to indicate changes in physical performance [45]. Nutritional biomarkers such as serum amino acids (including branched-chain amino acids) and serum albumin were assessed to reflect nutritional status [49]. Additional outcome variables included liver function enzymes (alanine aminotransferase, aspartate aminotransferase, and alkaline phosphatase), total bilirubin, and prothrombin time [49]. One (11%) study assessed disease severity based on the model for end-stage liver disease–sodium and Chronic Liver Failure Consortium acute decompensation scores to provide insight into disease progression and prognosis [43]. Finally, 1 (11%) prospective trial evaluated unplanned large-volume paracentesis frequency [43].

#### Psychosocial Outcomes

Two (22%) studies reported psychosocial outcomes, including QoL and cognitive function [45,47]. QoL was measured using the Chronic Liver Disease Questionnaire, EQ-5D-5L, and EQ-VAS [45]. Cognitive function was assessed in both studies using mobile-based tools designed to screen for covert hepatic encephalopathy [45,47].

#### Clinical Outcomes

Clinical outcomes focused on hospital admissions and mortality. Hospital admissions were described through admission frequency and length of stay [36,42,43,47], while mortality was assessed by comparing the number of deaths between groups [43,47].

#### Self-Management Outcomes

Self-management outcomes were assessed in 3 studies, covering 4 domains: physical activity behavior, nutritional behavior, self-management practice, and knowledge [45,46,49]. Physical activity was measured by tracking daily step counts using smartphone-linked devices [45,49]. Diet management was assessed in 2 studies using different approaches [45,46]. One study had participants record their daily protein intake through an application and complete 3-day food records, which were analyzed using dietary analysis software [45]. Another study used structured questionnaires that included items on dietary practices as part of a broader self-management assessment [46]. The same study evaluated self-management practice and knowledge through items addressing medication adherence, symptom monitoring, and understanding of disease management [46].

### Implementation Outcome Variables

Several studies reported implementation-related outcomes, categorized into feasibility and acceptability measures (n=6, 67%) and economic evaluations (n=1, 11%). These outcomes did not assess patient health status directly but rather evaluated the practicality, usability, and cost aspects of the interventions.

#### Feasibility and Acceptability

Six (67%) studies reported feasibility and acceptability outcomes using various indicators, including program completion rate, data transmission success, user satisfaction, and health care providers’ response rate to digital alerts [36,43-45,47,48]. Among these, 2 (22%) studies focused primarily on intervention feasibility and acceptability without evaluating direct clinical or health-related outcomes [44,48].

Additionally, 3 (33%) studies assessed usability and user acceptance using standardized instruments such as the Questionnaire for User Interaction Satisfaction and questionnaires based on the Technology Acceptance Model [44,46,48].

#### Economic Impact

One (11%) study evaluated economic impact by applying a model-based cost-effectiveness analysis [42]. The simulation modeled 100 hypothetical patients over a 6-month period, estimating potential health care cost savings associated with smartphone-based ascites management compared with standard care. The analysis included health care utilization costs such as hospital admissions, emergency visits, and outpatient procedures.

## Discussion

### Principal Findings

This scoping review identified and synthesized the current evidence on eHealth self-management interventions for adult patients with LC. Two key findings were identified. First, the interventions primarily focused on symptom monitoring, health behavior modification, health information provision, counseling and motivational support, alert-triggered responses, and reminder functions to promote patient self-management. Second, the study designs, definitions, and outcome measures showed significant heterogeneity, which limited the comparability and interpretation of findings.

Symptom monitoring was a central component of the eHealth self-management interventions, primarily aimed at detecting signs of clinical deterioration in LC. Studies commonly tracked both physical indicators (heart rate, blood pressure, body weight, body water composition, and abdominal circumference) and psychological indicators (cognitive function and subjective well-being). Among these, weight tracking was the most frequently implemented and was primarily used as a clinical marker of fluid retention due to complications such as ascites and peripheral edema [50]. This contrasts with its application in patients with MASLD, which generally focused on achieving and maintaining weight loss [51]. Treatment guidelines for patients with cirrhosis consider continuous weight measurement to be a valuable clinical indicator [52,53]. In particular, this measure can help determine the severity of edema when symptoms worsen and help manage obesity and related health problems, both of which are essential for assessing and improving the overall health of patients with cirrhosis.

Lifestyle modification strategies were also widely applied by the studies included in this review, typically combining dietary and physical activity components. Common dietary elements included sodium restriction and increased protein intake, while physical activity often involved walking programs or wearable-linked step tracking. Some studies added tools such as meal planners or dietary logs. However, these interventions only partially reflected clinical guidelines for LC, and key recommendations such as alcohol cessation, fluid intake control, and avoidance of hepatotoxic medications were rarely operationalized [52].

Compared with lifestyle interventions developed for other conditions, such as cardiovascular disease [54,55] or MASLD [51], which often include structured exercise prescriptions, habit formation techniques, and continuous coaching, the LC-targeted interventions in this review were less comprehensive and less systematically delivered [36,43,45-47,49]. Previous reviews of self-management programs for individuals with LC identified common components such as patient education, symptom monitoring, and coping strategies [26]. However, this review revealed that although eHealth self-management interventions incorporated these elements, they lacked a consistent framework or standardized structure. Additionally, although family member or caregiver involvement is known to enhance adherence, motivation, and long-term sustainability [56,57], the programs examined in this review were developed exclusively for individual patients without incorporating such outside participation. Therefore, future studies are required to develop eHealth self-management interventions that involve not only individual patients but also their families or caregivers and various health professionals.

This review also identified and categorized the various outcome domains reported in the included studies, which encompassed physical, clinical, behavioral, and psychosocial outcomes. Outcome variables and their measurement methods varied across studies. This diversity may reflect the exploratory nature of the current research on eHealth self-management interventions for people with cirrhosis. Similar issues have been noted in digital health research [58,59], where the lack of harmonization of results is a recognized limitation in cumulative learning and policy translation. These results highlight the need for greater standardization to support evidence synthesis on eHealth interventions for people with cirrhosis.

Most of the included studies were exploratory in nature, including pilot, feasibility, or usability designs. Although the definition and measurement methods of adherence were heterogeneous, several studies reported high completion and satisfaction rates, supporting the interventions’ feasibility and acceptability. However, only 1 study employed an RCT, and many lacked control groups, long-term follow-up, or theoretical frameworks. These limitations suggest that eHealth interventions for LC remain in an early phase of development. Future research should prioritize more rigorous, theory-informed designs with appropriate comparators, validated outcomes, and longer-term evaluation.

The studies included in this review used smartphone apps as eHealth tools; incorporated features such as disease education, automated and patient-reported data collection, medication reminders, and patient-provider communication; and provided alerts for timely intervention. eHealth self-management interventions help individuals set and achieve health goals related to weight management, diet, and physical activity, while allowing for remote symptom detection and effective problem management without requiring home visits [25,60,61]. Because of these benefits, these interventions are being tested for other chronic conditions. Study findings underscore the importance of eHealth interventions for patients who manage their diseases in outpatient settings rather than in hospital environments. Although comprehensive evidence is still developing, continued efforts should focus on applying eHealth interventions to empower asymptomatic or minimally compensated patients with LC to manage their health.

### Future Research Directions

Future research should focus on developing theory-based, standardized eHealth interventions tailored to the specific needs of patients with LC. These interventions must address key clinical challenges with clearly defined components. High-quality RCTs using validated and standardized measures are needed to evaluate short- and long-term outcomes [35]. Longitudinal studies will also be important for assessing sustained effects and patient engagement. Future interventions should better reflect clinical guidelines, incorporating not only diet but also recommendations such as alcohol cessation and fluid management [62]. The feasibility and scalability of these interventions should be tested in real-world outpatient settings, particularly in asymptomatic or compensated patients [63]. Finally, increased integration of structured family or caregiver roles and participation of multidisciplinary teams are required to enhance adherence and behavior maintenance to ensure eHealth intervention success.

### Strengths and Limitations

One strength of this review was its specific focus on eHealth interventions for patients with LC, an area that has received limited attention. By synthesizing the components of existing interventions, their delivery methods, and reported outcome domains, this review provides a structured overview of current evidence and highlights directions for future investigation. Notably, one included study evaluated the cost-effectiveness of eHealth interventions, indicating potential for health care cost savings [42]. However, this result was derived from a model-based simulation using hypothetical patient data rather than real-world clinical data. Therefore, the results should be considered exploratory, and further evidence is required to assess the effectiveness of the intervention. Nevertheless, the inclusion of economic analysis in this review is an important step forward in the evaluation of eHealth interventions for people with LC.

Despite these strengths, this review has several limitations. First, the small sample sizes of the included studies, along with the substantial heterogeneities in study designs and outcome measures, precluded quantitative synthesis. Second, the lack of validated measurement and inconsistent reporting undermined comparability. Third, most included studies employed exploratory designs without control groups or long-term follow-up. Finally, this review included only English- and Korean-language publications, potentially introducing language bias. These limitations underscore the early developmental stage of this research area and highlight the need for more rigorous, high-quality studies using standardized frameworks and comprehensive evaluation strategies.

### Conclusions

This scoping review systematically explored current evidence on eHealth self-management interventions for patients with LC. These interventions primarily focused on symptom monitoring, lifestyle modification, and counseling. However, the current body of literature is heterogeneous in scope and methodology. Many studies lacked standardized intervention frameworks and outcome measures, making it difficult to assess their effectiveness.

Future research should focus on advancing the development of eHealth self-management strategies and rigorously evaluating their effectiveness. Additionally, standardization of study designs and outcome reporting is critical for supporting evidence-based practice and enabling future systematic reviews and meta-analyses.

## Supplementary material

10.2196/68650 Multimedia Appendix 1 Search strategies.

10.2196/68650 Checklist 1 PRISMA-ScR checklist.

## Abbreviations

JBI: Joanna Briggs Institute
LC: liver cirrhosis
MASLD: metabolic dysfunction-associated steatotic liver disease
MeSH: Medical Subject Headings
PCC: Population, Concept, Context
PRISMA-ScR: Preferred Reporting Items for Systematic Reviews and Meta-Analyses Extension for Scoping Reviews
QoL: quality of life
RCT: randomized controlled trial
