# Supplementary material for: eHealth Self-Management Interventions for Patients With Liver Cirrhosis: Scoping Review
Source: J Med Internet Res. 2025 Sep 10;27:e68650. doi: 10.2196/68650 (PMC12422743; doi:10.2196/68650)
Supplement: Multimedia Appendix 1 [file jmir-v27-e68650-s001.pdf]

Supplement 1. Search strategies.

1. PubMed

Database	Keyword	Search	Query
**PubMed**	liver cirrhosis	#1	(liver cirrhosis[MeSH Terms]) OR (liver cirrhosis) OR (cirrho*) OR (liver disease[MeSH Terms]) OR (liver disease) OR (hepatic insufficiency[MeSH Terms]) OR (hepatic insufficiency) OR (hepatic failure) OR (liver failure[MeSH Terms]) OR (liver failure) OR (liver insufficiency) OR (liver fibrosis)
	self-management, lifestyle modification	#2	(healthy lifestyle[MeSH Terms]) OR (lifestyle modification) OR (lifestyle change) OR (self care[MeSH Terms]) OR (self care*) OR (self-care*) OR (self manage*) OR (self-manage*) OR (self efficacy[MeSH Terms]) OR (self efficac*) OR (self-efficac*) OR (personalized plan) OR (goal oriented) OR (personalized care plan) OR (management plan) OR (chronic disease management) OR (patient participation) OR (group, self help[MeSH Terms]) OR (self help) OR (self-help) OR (social support[MeSH Terms]) OR (social support) OR (patient cent*) OR (patient-cent*) OR (patient focus) OR (patient empower*) OR (patient train*) OR (patient instruct*) OR (patient educat*) OR ((behav*) AND ((therap*) OR (intervention*)))
	m-health	#3	(telemedicine[MeSH Terms]) OR (telemedicine) OR (telehealth) OR (tele-health) OR (mobile health units[MeSH Terms]) OR (mobile health units) OR (mhealth) OR (m-health) OR (cell phone[MeSH Terms]) OR (cell phone) OR (smartphone[MeSH Terms]) OR (smartphone) OR (satellite phone) OR (smartphone-based) OR (computers, handheld[MeSH Terms]) OR (tablet computer) OR (tablet pc) OR (computers[MeSH Terms]) OR (computers) OR (mobile application) OR (mobile app) OR (ehealth) OR (e-health) OR (internet-based intervention[MeSH Terms]) OR (internet based) OR (internet-based) OR (web based) OR (web-based) OR (text messaging[MeSH Terms]) OR (text messag*)
	Language	#4	(English[Language]) OR (Korean[Language])
	Date	#5	("2013/01/01"[Date - Publication] : "2025/06/30"[Date - Publication])
			**#1 AND #2 AND #3 AND #4 AND #5**

2. Embase

**Embase**	liver cirrhosis	#1	('liver cirrhosis') OR (cirrho*) OR ('liver disease') OR (hepatic insufficiency) OR (hepatic failure) OR ('liver failure') OR (‘liver insufficiency’) OR (‘liver fibrosis’)
	self-management, lifestyle modification	#2	('healthy lifestyle') OR (‘lifestyle modification’) OR (lifestyle change) OR ('self-care*') OR (‘self care*’) OR (‘self-manage*’) OR ('self manage*') OR ('self-efficac*') OR (‘self efficacy’) OR (‘personalized plan’) OR (‘personalized care’) OR (‘goal-oriented’) OR (‘goal oriented’) OR (‘management plan’) OR (‘chronic disease management’) OR ('patient participation') OR ('self-help') OR ('self help') OR ('social support') OR (‘patient-cent*’) OR ('patient cent*') OR (patient focus) OR ('patient empowerment') OR (patient train*) OR (patient instruct*) OR ('patient education') OR (patient educat*) OR ('behavior therapy') OR (((behav*)) AND ((therap*) OR (intervention*)))
	m-health	#3	('telemedicine') OR ('tele-health') OR ('telehealth') OR (‘mobile health unit’) OR (‘m-health’) OR ('m health') OR ('mobile phone') OR (‘cell phone’) OR ('smartphone') OR (‘satellite phone’) OR ('tablet computer') OR (‘tablet pc’) OR ('computer') OR ('mobile application') OR (‘mobile app’) OR (‘e-health’) OR ('ehealth') OR (‘web-based’) OR (‘web based’) OR (‘internet-based’) OR (‘internet based’) OR (text messag*)
	Language	#4	([english]/lim OR [korean]/lim)
	Date	#5	[2013-2025]/py
			**#1 AND #2 AND #3 AND #4 AND #5**

3. Cochrane library

**Cochrane library**	liver cirrhosis	#1	(MeSH descriptor: [Liver Cirrhosis]) OR (liver cirrhosis) OR (cirrho*) OR (MeSH descriptor: [Liver Diseases]) OR (liver disease) OR (MeSH descriptor: [Hepatic Insufficiency]) OR (hepatic insufficiency) OR (hepatic failure) OR (MeSH descriptor: [Liver Failure]) OR (liver failure) OR (liver insufficiency) OR (liver fibrosis)
	self-management, lifestyle modification	#2	(MeSH descriptor: [Healthy Lifestyle]) OR (healthy lifestyle) OR (lifestyle modification) OR (lifestyle change) OR (MeSH descriptor: [Self Care]) OR (self care*) OR (self-care*) OR (MeSH descriptor: [Self-Management]) OR (self manage*) OR (self-manage*) OR (MeSH descriptor: [Self Efficacy]) OR (self efficac*) OR (self-efficac*) OR (personalized plan) OR (goal oriented) OR (personalized care plan) OR (management plan) OR (chronic disease management) OR (MeSH descriptor: [Patient Participation]) OR (patient participation) OR (self help) OR (self-help) OR (MeSH descriptor: [Social Support]) OR (social support) OR (patient cent*) OR (patient-cent*) OR (patient focus) OR (patient empower*) OR (patient train*) OR (patient instruct*) OR (MeSH descriptor: [Patient Education as Topic]) OR (patient educat*) OR ((behav*) AND ((therap*) OR (intervention*)))
	m-health	#3	(MeSH descriptor: [Telemedicine]) OR (telemedicine) OR (telehealth) OR (tele-health) OR (MeSH descriptor: [Mobile Health Units]) OR (mobile health units) OR (mhealth) OR (m-health) OR (MeSH descriptor: [Cell Phone]) OR (cell phone) OR (MeSH descriptor: [Smartphone]) OR (smartphone) OR (satellite phone) OR (smartphone-based) OR (smartphone based) OR (MeSH descriptor: [Computers, Handheld]) OR (tablet computer) OR (tablet pc) OR (MeSH descriptor: [Computers]) OR (computers) OR (MeSH descriptor: [Mobile Applications]) OR (mobile application) OR (mobile app) OR (ehealth) OR (e-health) OR (MeSH descriptor: [Internet-Based Intervention]) OR (internet based) OR (internet-based) OR (web based) OR (web-based) OR (MeSH descriptor: [Text Messaging]) OR (text messag*)
	Language	#4	(English:la) OR (Korean:la)
	Date	#5	With Cochrane Library publication date Between Jan 2013 and June 2025
			**#1 AND #2 AND #3 AND #4 AND #5**

4. CINAHL

**CINAHL**	liver cirrhosis	#1	(MH "Liver Cirrhosis+") OR (MH "Liver Diseases+") OR (MH "Liver Failure, Acute") OR (TX liver cirrhosis) OR (TX cirrho*) OR (TX liver disease) OR (TX hepatic insufficiency) OR (TX hepatic failure) OR (TX liver failure) OR (TX liver insufficiency) OR (TX liver fibrosis)
	self-management, lifestyle modification	#2	(MH "Life Style Changes") OR (MH "Self Care+") OR (MH "Self-Management") OR (MH "Self-Efficacy") OR (MH "Support, Social+") OR (MM "Patient Centered Care") OR (TX healthy lifestyle) OR (TX lifestyle modification) OR (TX lifestyle change) OR (TX self care*) OR (TX self-care*) OR (TX self manage*) OR (TX self-manage*) OR (TX self efficac*) OR (TX self-efficac*) OR (TX personalized plan) OR (TX personalized care) OR (TX goal oriented) OR (TX management plan) OR (TX chronic disease management) OR (TX patient participation) OR (TX self help) OR (TX self-help) OR (TX social support) OR (TX patient cent*) OR (TX patient-cent*) OR (TX patient centered care) OR (TX patient focus) OR (TX patient empower*) OR (TX patient train*) OR (TX patient instruct*) OR (TX patient educat*) OR (((TX behav*)) AND ((TX therap*) OR (TX intervention*)))
	m-health	#3	(MH "Telemedicine+") OR (MM "Mobile Health Units") OR (MH "Cellular Phone+") OR (MM "Smartphone") OR (MM "Mobile Applications") OR (MM "Internet-Based Intervention") OR (MH "Text Messaging+") OR (TX telemedicine) OR (TX telehealth) OR (TX tele-health) OR (TX mobile health units) OR (TX mhealth) OR (TX m-health) OR (TX cell phone) OR (TX smartphone) OR (TX satellite phone) OR (TX smartphone-based) OR (TX computer) OR (TX tablet computer) OR (TX tablet pc) OR (TX mobile application) OR (TX mobile app) OR (TX mobile app) OR (TX ehealth) OR (TX e-health) OR (TX internet based) OR (TX internet-based) OR (TX web based) OR (TX web-based) OR (TX text messag*)
	Language	#4	(LA English) OR (LA Korean)
	Date	#5	Limiters – Publication Date: 20130101-20250630
			**#1 AND #2 AND #3 AND #4 AND #5**

5. Web of Science

**Web of Science**	liver cirrhosis	#1	ALL=(liver cirrhosis) OR ALL=(cirrho*) OR ALL=(liver disease) OR ALL=(hepatic insufficiency) OR ALL=(hepatic failure) OR ALL=(liver failure) OR ALL=(liver insufficiency) OR ALL=(liver fibrosis)
	self-management, lifestyle modification	#2	ALL=(healthy lifestyle) OR ALL=(lifestyle modification) OR ALL=(lifestyle change) OR ALL=(self care*) OR ALL=(self-care*) OR ALL=(self manage*) OR ALL=(self-manage*) OR ALL=(self efficac*) OR ALL=(self-efficac*) OR ALL=(personalized plan) OR ALL=(goal oriented) OR ALL=(personalized care plan) OR ALL=(management plan) OR ALL=(chronic disease management) OR ALL=(patient participation) OR ALL=(self help) OR ALL=(self-help) OR ALL=(social support) OR ALL=(patient cent*) OR ALL=(patient-cent*) OR ALL=(patient focus) OR ALL=(patient empower*) OR ALL=(patient train*) OR ALL=(patient instruct*) OR ALL=(patient educat*) OR ((ALL=(behav*)) AND (ALL=(therap*) OR ALL=(intervention*)))
	m-health	#3	ALL=(telemedicine) OR ALL=(telehealth) OR ALL=(tele-health) OR ALL=(mobile health units) OR ALL=(mhealth) OR ALL=(m-health) OR ALL=(cell phone) OR ALL=(smartphone) OR ALL=(satellite phone) OR ALL=(smartphone-based) OR ALL=(tablet computer) OR ALL=(tablet pc) OR ALL=(computer) OR ALL=(mobile application) OR ALL=(mobile app) OR ALL=(ehealth) OR ALL=(e-health) OR ALL=(internet based) OR ALL=(internet-based) OR ALL=(web based) OR ALL=(web-based) OR ALL=(text messag*)
	Language	#4	LA = (English OR Korean)
	Date	#5	DOP = (2013-01-01 / 2025-06-30)
			**#1 AND #2 AND #3 AND #4 AND #5**
